# Transcriptome-Wide Profiling and Expression Analysis of Diploid and Autotetraploid *Paulownia tomentosa* × *Paulownia fortunei* under Drought Stress

**DOI:** 10.1371/journal.pone.0113313

**Published:** 2014-11-18

**Authors:** Enkai Xu, Guoqiang Fan, Suyan Niu, Zhenli Zhao, Minjie Deng, Yanpeng Dong

**Affiliations:** 1 Institute of Paulownia, Henan Agricultural University, Jinshui Area, Zhengzhou, Henan, P.R. China; 2 College of Forestry, Henan Agricultural University, Jinshui Area, Zhengzhou, Henan, P.R. China; National Taiwan University, Taiwan

## Abstract

Paulownia is a fast-growing deciduous hardwood species native to China, which has high ecological and economic value. In an earlier study, we reported ploidy-dependent differences in Paulownia drought tolerance by the microscopic observations of the leaves. Autotetraploid Paulownia has a higher resistance to drought stress than their diploid relatives. In order to obtain genetic information on molecular mechanisms responses of Paulownia plants to drought, Illumina/Solexa Genome sequencing platform was used to *de novo* assemble the transcriptomes of leaves from diploid and autotetraploid *Paulownia tomentosa* × *Paulownia fortunei* seedlings (PTF2 and PTF4 respectively) grown under control conditions and under drought stress and obtained 98,671 nonredundant unigenes. A comparative transcriptome analysis revealed that hundreds of unigenes were predicted to be involved mainly in ROS-scavenging system, amino acid and carbohydrate metabolism, plant hormone biosynthesis and signal transduction, while these unigenes exhibited differential transcript alteration of the two accessions. This study provides a comprehensive map of how *P. tomentosa* × *P. fortunei* responds to drought stress at physiological and molecular levels, which may help in understanding the mechanisms involve in water-deficit response and will be useful for further study of drought tolerance in woody plants.

## Introduction

Plants are constantly exposed to a wide range of environmental stresses such as drought, high salt, heat, and extremes of temperature. Water scarcity is one of the major environmental stresses that causes substantial losses in many plants [1]. It affects every aspect of plant growth, modifying anatomy, morphology, physiology, and biochemistry, which negatively affects plant growth and productivity [2].

Global climate change will likely reduce water availability further increasing the need for drought tolerant plants [3]. To adapt to drought stress, trees have evolved complex mechanisms based on modifications in metabolites, gene expression, and proteins. Clearly, drought tolerance is a complex trait that is regulated by multiple genes. Based on their putative biological functions in the drought response process, drought-responsive genes have been classified into two groups: (1) genes encoding proteins that defend the cells from the effects of water-deficit, for example, proteins that combat the accumulation of solutes (e.g., enzymes for biosynthesis of osmolytes like proline, betaine, sugars), passive and active transport systems across membranes (e.g., water channel proteins and membrane transporters), proteins that protect and stabilize cell structures from damage by reactive oxygen species (e.g., detoxification enzymes such as catalase and superoxide dismutase), and other proteins that protect the macromolecules (e.g., late embryogenesis-abundant (LEA) proteins and osmotin) [4]–[6]; and (2) genes encoding proteins that regulate stress signal transduction and alter gene expression (e.g., transcription factors, protein kinases, protein phosphatases, proteinases, and phytohormones) [7], [8].

The underlying mechanisms of the drought stress response in plants have been investigated using various genomic and genetic tools. Cohen et al. [9] assessed the genotype-dependent component of a drought-induced transcriptome response in two poplar genotypes with different drought tolerances using a multi-species designed microarray. Zheng et al. [10] compared genome-wide gene expression profiles between drought-tolerant and drought-sensitive lines of maize during drought stress by microarray.

High-throughput sequencing is a powerful and cost-efficient tool that has been used effectively in many areas, including the study of the transcriptomes of plants under biotic and abiotic stresses. Over 24,000 drought-responsive genes were identified from *Populus euphratica* leaves using a Roche/454 platform [11]. The transcriptomes of 22 different developmental stage tissues from *Cicer arietinum L.* were sequenced using a Illumina/Solexa system, which revealed thousands of drought-responsive genes [12]. Using Illumina/Solexa sequencing, Peng et al. [13] reported differences in the transcriptome levels between male and female *Populus yunnanensis* in response to drought stress.

Paulownia is a species of tree that is native to China, where it has been cultivated for over 2000 years. This species has also been planted in many other countries and can now be found throughout the world, except Antarctica [14]. Paulownia is an adaptable and fast-growing deciduous tree, which provides raw material for manufacturing furniture, plywood, and musical instruments. It has a unique biological characteristic of late leaf emergence and fall, a deep root system, and sparse branching; therefore, exemplary areas of agroforestry have been built on the broad plains in North-Central China [15]. Paulownia has a very large leaf area with a high transpiration rate; therefore, sufficient moisture is very important for Paulownia growth. *Paulownia tomentosa* × *Paulownia fortunei* is grown mainly in hilly loess areas or in salinized soil [16] where the annual precipitation is below 500 mm. *P. tomentosa* × *P. fortunei* grow quite well without artificial irrigation because of their inherent ability to withstand drought. Therefore, *P. tomentosa* × *P. fortunei* is an ideal species in which to study the mechanisms of drought tolerance.

Polyploidization of chromosomes is thought to be one of the most important mechanisms for species evolution [17]. Polyploidy usually changes plant morphological and anatomical characteristics introducing, for example, superior breast-height diameter, volume, leaf, fruit, and stoma [18]–[20]. Polyploidy also changes the physiology, biochemistry, and gene expression in plants [17], [21], [22]. Several studies have reported that polyploids exhibit higher stress resistance compared with their corresponding diploid relatives [23]–[25].

To benefit from the possible value of polyploidy, in 2010, an autotetraploid *P. tomentosa* × *P. fortunei* (2*n* = 4*x* = 80) was induced from the leaves of diploid *P. tomentosa* × *P. fortunei* (2*n* = 2*x* = 40) using colchicines (an established system of *in vitro* plantlet regeneration) and a detailed physiological study of diploid and autotetraploid plants was conducted [26].

Drought tolerance varies considerably between diploid and autotetraploid Paulownia. In an earlier paper, we studied diploid and autotetraploid Paulownia plants for their drought and cold tolerance by microscopic observations of the leaves [27]. However, little is known about the molecular mechanisms of diploid and autotetraploid Paulownia under drought conditions.

In the present study, to reveal the transcriptome-wide response to drought stress in *P. tomentosa* × *P. fortunei* and to investigate diploid and autotetraploid differences in leaf transcriptome remodeling during drought stress, we analyzed the gene expression profiles of diploid and autotetraploid seedlings using an Illumina/Solexa Genome Analyzer IIx (GAIIx) platform. To the best of our knowledge, this is the first comprehensive analysis of differentially expressed genes in drought-stressed *P. tomentosa* × *P. fortunei*. A comparison of the diploid and autotetraploid transcriptomes revealed differences and similarities, which helped identify stress-related candidate genes and gene regulatory networks for future screening. The acquired information will provide new insights into the molecular mechanisms involved in the *P. tomentosa* × *P. fortunei* response to drought stress.

## Materials and Methods

### Plant material

Uniformly grown tissue culture seedlings of diploid *P. tomentosa* × *P. fortunei* (PTF2) and tetraploid *P. tomentosa* × *P. fortunei* (PTF4) were collected from the Institute of Paulownia, Henan Agricultural University, Zhengzhou, Henan Province, China and cultivated in nutrition blocks containing ordinary garden soil for 30 days. Samples with the same crown size and height were then planted in plastic pots (20 cm in diameter at the bottom and 20 cm deep) containing ordinary garden soil, one for each plant. The plants were housed randomly in an outdoor nursery for 50 days before being subjected to stress treatment. Twelve plants from each accession (PTF2 and PTF4) were obtained, including three controls and nine plants for water-deficit treatment (divided into three groups, each group contained three individual biological replicates).

The seedlings were subjected to drought conditions in a water-controlled experiment according to the method of Moumeni et al. [28]; the controls were watered to field capacity every day. For drought treatment, the seedlings of the two accessions were subjected to mild (5 days without irrigation, 55% relative soil water content), moderate (15 days without irrigation, 40% relative soil water content), and severe (20 days without irrigation, 25% relative soil water content) drought stress. The mature, fully expanded leaves (second leaf from the apex) were sampled and mixed in equal numbers from each plant in each of the replicate groups. The leaf tissue was frozen in liquid nitrogen and then stored at −80°C for later use.

### Physiological measurements

Phenotypic and physiological characteristics of the PTF2 and PTF4 seedlings were measured under the control, mild, moderate, and severe drought stresses.

Physiological measurements were made between 8.30 am and 11.00 am on single, fully expanded leaves (third and fourth leaves from the terminal bud of a twig) immediately after excision. The measurements were taken on three replicates of the leaves from the same plants.

The percent relative water content of the leaf was calculated as (fresh weight - dry weight)/(turgid weight - dry weight) × 100 [29]. Chlorophyll content was measured according to a described method previously [30] and calculated as described by Arnon [31]. The malondialdehyde concentration was measured using the thiobarbituric acid method as described by Hodges et al. [32]. Relative electrical conductivity was measured as described previously [33]. Superoxide dismutase activity was determined using the nitroblue tetrazolium method reported by Dhindsa et al. [34]. Values were expressed in units/gmHb [35]. Total soluble protein content was measured following the procedure of Guy et al. [36], and the protein concentration was measured as described by Bradford [37] using bovine serum albumin (Sigma, USA) as the standard. Proline content was measured using the acid ninhydrin method of Bates et al. [38]. Soluble sugar content was measured using the anthrone colorimetry method described by Irigoyen et al. [39].

### Statistical analysis

Analyses of variance (ANOVA) of the parameters were performed using the MSTAT-C computer program (MSTAT Development Team, Michigan State University, 1989) and SPSS (v. 16.5). The mean values of each of the measured traits were compared using the least significance difference (LSD) test at a 5% level of probability according to Snedecor and Cochran [40].

### RNA extraction and double-stranded cDNA synthesis

The PTF2 and PTF4 seedlings from the severe drought-stressed group were chosen for sequencing based on the physiological measurements (see the results section for details).

Approximately 8 mg of leaves from the diploid control (well-watered PTF2) and the severe drought-treated PTF2, and 8 mg of leaves from the tetraploid control (well-watered PTF4 control) and the severe drought-treated PTF4 in three replications were used for total RNA extraction.

Total RNA was extracted from the four samples with TRIzol reagent (Invitrogen, Carlsbad, CA). RNA was purified using RNeasy MiniElute Cleanup Kit (Qiagen, Valencia, CA) according to the manufacturer’s protocol. Magnetic beads with Oligo (dT) were used to isolate poly-(A) mRNA from the total RNA. The mRNA was mixed with fragmentation buffer and fragmented into short fragments. The first-strand cDNA was synthesized with SuperScript II reverse transcriptase (Life Technologies, Carlsbad, CA), and DNA polymerase I and RNase H were then used to synthesize the second-strand cDNA. The short cDNA fragments were purified and dissolved in EB buffer for end reparation and addition of a single nucleotide A (adenine). The short fragments were then connected to adapter sequences. Suitable fragments were selected as templates for PCR amplification. The cDNA libraries were qualified using a 2100 Bioanalyzer (Agilent Technologies, Inc., Santa Clara, CA), then sequenced on a Illumina Genome Analyzer IIx (GAIIx) platform (Illumina, San Diego, CA) by Beijing Genomics Institute (BGI)-Shenzhen, Shenzhen, China, following the manufacturer’s standard cBot and sequencing protocols.

### Assembly and annotation

The raw image data were transformed by base calling into sequence data (raw reads). Raw reads that contained adaptor sequences, ambiguous reads, or low-quality reads were removed using DynamicTrim in the SolexaQA package (http://solexaqa.sourceforge.net/) to create clean reads. The clean reads used in this publication have been deposited in the NIH Short Read Archive (SRA) database (http://www.ncbi.nlm.nih.gov/sra) under accession number SRP041407. The quality sequences were assembled into unigenes using the Trinity program [41]. Unigenes from multiple samples from the same accession were further processed by removing redundancy and splicing, and then non-redundant unigenes without gaps were clustered using the TIGR Gene Indices clustering tools (TGICL) [42]. The unigene sequences were annotated by aligning them against the NCBI non-redundant protein sequence database (Nr) (http://www.ncbi.nlm.nih.gov/), Swiss-Prot (http://www.expasy.ch/sprot/), Cluster of Orthologous Groups (COG) (http://www.ncbi.nlm.nih.gov/cog/), and the Kyoto Encyclopedia of Genes and Genomes (KEGG) (http://www.genome.jp/kegg/) using BLASTX (E-value <1.0E-5), and then against the NCBI nucleotide sequence database (Nt) using BLASTN (E-value <1.0E-5).

The best alignments were used to annotate the unigenes and to decide the coding region and sequence direction of the unigenes. Non-annotated unigenes with no matches in any of the searched databases were scanned using ESTScan [43] to predict the sequence (5′–3′) direction and to predict the coding region to obtain the translated amino sequence. Gene Ontology (GO) functional classification of the unigenes was performed using Blast2GO [44] and WEGO software [45]. Pathway annotations were obtained by aligning the unigenes with the KEGG database [46].

### Unigene expression difference analysis

Unigene expression in each library was calculated using the FPKM (fragments per kb per million fragments) method [47] as:
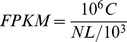
where *FPKM* is the expression of unigene A, C is the number of fragments that aligned uniquely to unigene A, *N* is the total number of fragments that aligned uniquely to all the unigenes, and *L* is the number of bases in the coding sequence of unigene A.

Differentially expressed unigenes (DEUs) between the PTF2 and PTF4 transcriptomes were identified using the rigorous algorithm described by Audic et al. [48].

To screen the DEUs, the p-value threshold in multiple hypothesis testing and analyses was determined by manipulating the false discovery rate (FDR) value [49]. A FDR ≤0.001 and absolute value of the |log2Ratio| >2 were used as the threshold to determine the significance of the differences in gene expression [49]. The DEUs were then subjected to GO functional and KEGG Pathway analyses.

### GO and pathway enrichment analysis

GO functional enrichment analysis was performed to classify the GO functional annotations for the DEUs. First, all the DEUs were mapped to terms in the GO database (http://www.geneontology.org/) and the gene numbers for each GO term were calculated to obtain a gene list and the gene numbers for every mapped GO term. The GO enrichment analysis was performed by applying the hypergeometric test to find significantly enriched GO terms in the DEUs compared with the whole transcriptome background of *P. tomentosa* × *P. fortunei*. The p-value was calculated as:
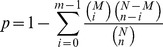
where N is the total number of genes with GO annotation; n is the number of DEUs in N; M is the total number of all genes that were annotated to a certain GO term; and m is the number of DEUs in M. After applying the Bonferroni correction to the calculated p-value we chose a corrected p-value of ≤0.05 as the threshold, and GO terms with p≤0.05 were defined as significantly enriched GO terms for the DEUs.

Similarly, a pathway enrichment analysis to identify significantly enriched biochemical or signal transduction pathways associated with the DEUs was performed by comparing the pathway annotations with the whole transcriptome background of *P. tomentosa* × *P. fortunei*. A Q-value, defined as the FDR, was used to correct the p-value. After multiple test corrections, we chose a Q-value of ≤0.05 to identify significantly enriched pathways for the DEUs.

### Real-time quantitative PCR analysis of potential drought response DEUs

A real-time quantitative PCR (qRT-PCR) analysis was conducted on selected DEUs to validate the Illumina paired-end sequencing results. *P. tomentosa* × *P. fortunei* leaves collected from the severe drought and control PTF2 and PTF4 plants were used to extract total RNA. The first-strand cDNA was synthesized from 2 mg total RNA with the iScriptc DNA synthesis kit (Bio-Rad, Hercules, CA) according to the manufacturer’s instructions.

The specific primers used for the real-time PCR assay were designed using Beacon Designer, version 7.7 (Premier Biosoft International, Ltd., Palo Alto, CA) and are listed in Table S1. The cDNA was amplified using a CFX96TM Real-Time System (Bio-Rad) with So Fast Eva Green Supermix (Bio-Rad) according to the manufacturer’s instructions. PCR mixtures with a final volume of 20 µL contained 10 µL SYBR Green PCR mix, 1 µL cDNA, 0.4 µM forward primer, 0.4 µM reverse primer, and 7.0 µL SteriLe ddH_2_O. The PCR cycles were as follows: 95°C for 1 min, followed by 40 cycles of 95°C for 10 s, and 55°C for 15 s. Relative expression levels of the genes were calculated using the 2^−ΔΔCt^ method and normalized with 18S rRNA from *P. tomentosa* × *P. fortunei.*

## Results

### Physiological characterization of P. tomentosa × P. fortunei under drought stress

The phenotypic and physiological responses of PTF2 and PTF4 to water deficit were assessed using seedlings exposed to progressive water loss for about three weeks.

**Figure 1:**
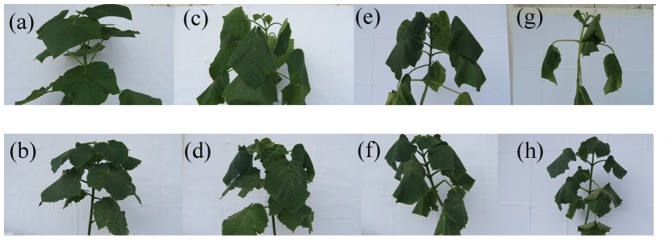
Physiological feature of diploids and autotetraploid in response to drought. (a) PTF2W, well-watered diploid, 75% relative soil water content; (b) PTF4W, well-watered tetraploid, 75% relative soil water content; (c) PTF2T-1, 5-day drought treated diploid, 55% relative soil water content; (d) PTF4T-1, 5-day drought treated tetraploid, 55% relative soil water content; (e) PTF2T-2, 15-day drought treated diploid, 40% relative soil water content; (f) PTF4T-2, 15-day drought treated tetraploid, 40% relative soil water content; (g) PTF2T-3, 20-day drought treated diploid, 25% relative soil water content; (h) PTF4T-3, 20-day drought treated tetraploid, 25% relative soil water content.

There were no significant differences in stress phenotype between the PTF2 and PTF4 under the well-watered conditions (Figure 1a and 1b). The leaves of the PTF2 seedlings drooped after five days of drought stress (Figure 1c), whereas the leaves of the PTF4 seedlings remained green and turgid (Figure 1d). After 15 days, the PTF2 plants showed stunted growth and had leaves that were paler and more wilted compared with the PTF4 plants (Figure 1e and 1f); after 20 days, the PTF2 and PTF4 plants both showed distinct effects of drought stress. However, the PTF4 plants showed much better development, less wilting, and higher biomass compared with the PTF2 plants (Figure 1 g and 1 h).

In general, the PTF4 seedlings maintained markedly higher growth vigor under the drought stress conditions than the PTF2 seedlings.

### Changes in physiological parameters in PTF2 and PTF4 plants in response to drought

**Figure 2:**
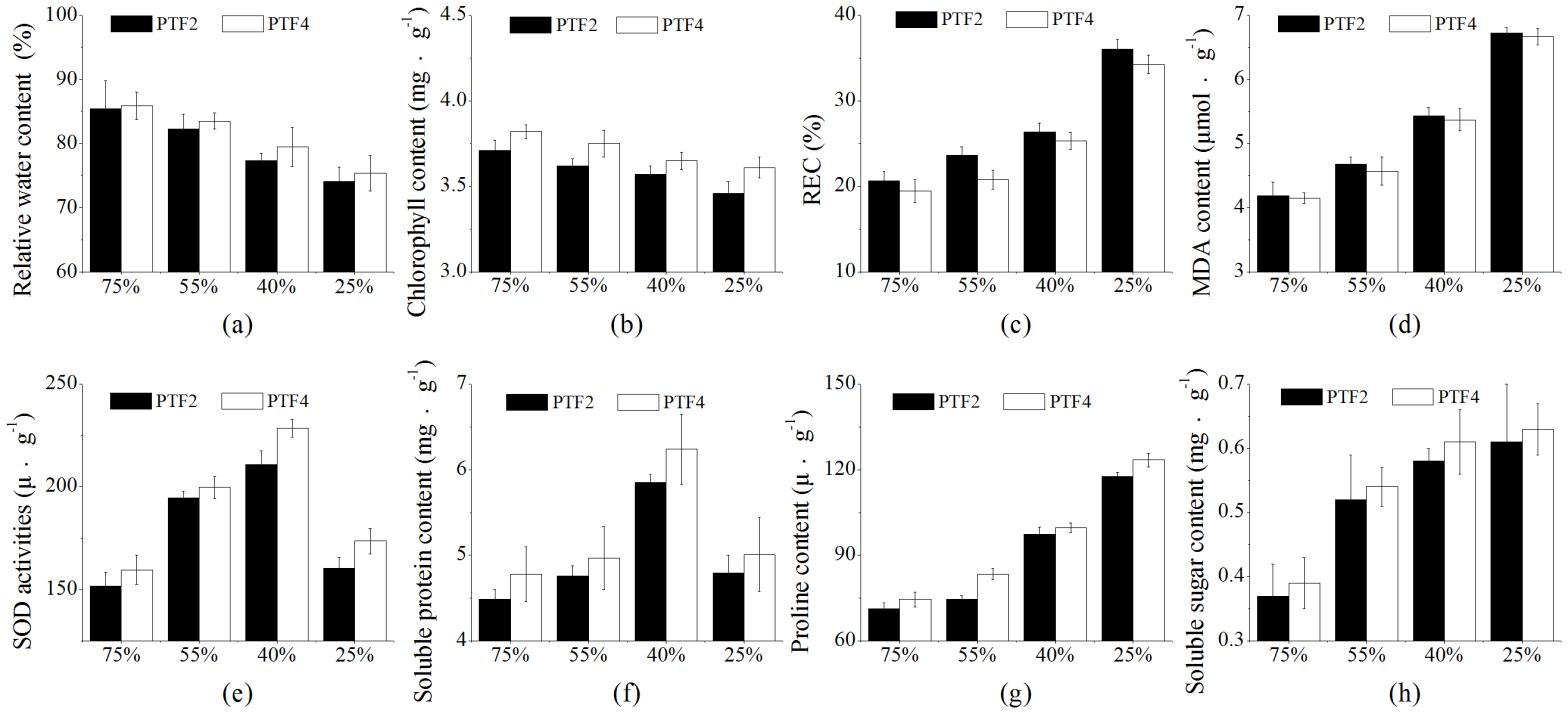
Effects of drought stress on *P. tomentosa* × *P. fortunei* physiology. PTF2 represents diploid *P. tomentosa* × *P. fortunei*, PTF4represents autotetraploid *P. tomentosa* × *P. fortunei*, 75%, 55%, 40% and 25% relative soil water contents were used. (a) Effect of drought stress on leaf relative water content;(b) Effect of drought stress on leaf chlorophyll content; (c) Effect of drought stress on leaf relative electrical conductivity; (d) Effect of drought stress on leaf malondialdehyde (MDA) content; (e) Effect of drought stress on leaf superoxide dismutase (SOD) activities; (f) Effect of drought stress on leaf soluble protein content; (g) Effect of drought stress on leaf proline content; (h) Effect of drought stress on leaf soluble sugar content.

Drought stress significantly inhibited the relative water content (RWC) and chlorophyll content of the plants. The RWC of PTF2 was similar to that of PTF4 under well-watered conditions. The RWC of both accessions declined with the increase of the drought, as characterized by their significantly lower RWC compared with the corresponding controls (well-watered), particularly under the severe drought treatment. However, after 20 days of drought, the PTF2 plants showed a sharp decrease in RWC (13.25%) compared with the RWC (12.23%) of the PTF4 plants (Figure 2a). PTF2 and PTF4 were recorded the highest chlorophyll content under well-watered conditions while, during the drought treatment, the chlorophyll content significantly decreased in both accessions. The chlorophyll content also obviously showed difference between PTF2 and PTF4 plants, and the PTF4 seedlings always contained higher levels of total chlorophyll (chlorophyll a and b) than the PTF2 seedlings under drought stress (Figure 2b).

Relative electrical conductivity (REC) and malondialdehyde (MDA) content showed overall increased tendencies in the PTF2 and PTF4 plants under drought stress. However, after 20 days of drought stress the REC and MDA values increased significantly in both accessions, whereas the REC and MDA in the PTF2 plants were always at much higher levels than in the PTF4 plants over the entire treatment process (Figure 2c and 2d).

Compared to the control conditions, both the PTF2 and PTF4 plants under mild and moderate drought conditions showed a substantial increase in superoxide dismutase (SOD) activity and soluble proteins content, and then decreased dramatically under severe drought stress, whereas the values of these two traits were higher in both PTF2 and PTF4 plants under severe drought than under well-watered conditions. Besides, SOD activity and soluble proteins content measurements showed striking differences between the two accessions under the same drought treatments. In particular, PTF4 showed higher SOD activity and soluble proteins content than PTF2 at the end of the 20-day experiment (Figure 2e and 2f).

Additionally, the proline and soluble sugar contents tended to increase as the drought strengthened in the two accessions. During the different drought treatments, the proline and soluble sugar contents in PTF4 increased significantly, but in PTF2 they showed relatively little changes. Overall, the PTF4 plants showed much higher levels of the proline and soluble sugar contents compared with the PTF2 plants (Figure 2 g and 2 h).

### Paired-end sequencing and de novo assembly of the P. tomentosa × P. fortunei reads

To understand the molecular mechanisms of drought tolerance in *P. tomentosa* × *P. fortunei*, four leaf cDNA libraries, two for the well-watered controls (PTF2W, PTF4W) and two for the severe drought-treated plants (PTF2T, PTF4T), were constructed and sequenced on the GAIIx platform. Over 291 million raw reads with a mean length of 110 nt, were generated from the four libraries; approximately 73 million reads from each sample. After removing adapter sequences, duplication sequences, and ambiguous and low-quality reads, 264 (23.78 Gb) million clean reads remained. The Q20 (sequencing error rate, 1%) percentage, N percentage, and GC content were 97.43%, 0%, and 49.04%, respectively (Table 1). The *de novo* assembly of the clean reads using Trinity generated 599,908 contigs without gaps, with an average length of 304 nt and an N50 of 471 nt. After clustering using the TGICL software, a total of 98,671 nonredundant unigenes were produced, including 47,582 clusters and 51,089 singletons. The total length of the unigenes was 96,940,229 nt, with a mean length of 982 nt and an N50 of 1626 nt (Table 2); 63,015 (63.86%) unigenes were longer than 500 nt and 38,134 (38.65%) were longer than 1,000 nt. The length distribution of the unigenes is shown in Figure S1. None of the unigenes had gaps, indicating that the sequences were of high quality. All the assembled unigenes were aligned against the Nr, Swiss-Prot, KEGG, and COG protein databases, and a total of 70,524 coding sequences (CDSs) were predicted; 67,605 CDSs were inferred using BLASTX hits and 2,919 were assigned using ESTScan.

**Table 1:** Summary of the sequence assembly after Illumina sequencing.

Samples	Total Raw Reads	Total Clean Reads	Total Clean Nucleotides (nt)	Q20 percentage	N percentage	GC percentage
PTF2W	72,147,152	65,657,050	5,909,134,500	97.50%	0.00%	46.42%
PTF2 T	72,176,810	65,227,994	5,870,519,460	97.42%	0.00%	46.07%
PTF4W	76,705,742	69,169,960	6,225,296,400	97.43%	0.00%	46.90%
PTF4 T	70,571,228	64,168,586	5,775,172,740	97.37%	0.00%	47.29%

**Table 2:** Summary statistics of contig and unigene in four libraries from *P. tomentosa* × *P. fortune*.

	Sample	Total Number	Total Length(nt)	Mean Length(nt)	N50	Total Consensus Sequences	Distinct Clusters	Distinct Singletons
Contig	PTF2W	144,755	45,259,057	313	508	–	–	–
	PTF2 T	161,799	47,608,475	294	441	–	–	–
	PTF4W	143,358	45,001,926	314	494	–	–	–
	PTF4 T	149,996	44,356,767	296	442	–	–	–
Unigene	PTF2W	78,883	59,470,771	754	1332	78,883	32,443	46,440
	PTF2 T	89,241	69,693,869	781	1429	89,241	36,185	53,056
	PTF4W	81,816	57,290,184	700	1209	81,816	31,763	50,053
	PTF4 T	79,025	55,450,884	702	1240	79,025	30,515	48,510
	All	98,671	96,940,229	982	1626	98,671	47,582	51,089

All the statistics showed that the sequencing of the *P. tomentosa* × *P. fortunei* transcriptomes was of high depth and that high quality reads were produced.

### Annotation and functional analysis of the unigenes

The BLAST searches against the various databases yielded a total of 67,326 (68.23%) unigenes that found matches against the Nr database, followed by Nt (58,508, 59.30%), GO (55,413, 56.16%), Swiss-Prot (43,851, 44.44%), KEGG (41,206, 41.76%), and COG (28,165, 28.55%) (Table S2).

Overall, 70,100 (71.04%) unigenes shared similarities with entries in one or more of the six databases. No matches were found for 28,571 of the unigene sequences in any of these databases (E-value >10-5), suggesting that some of these unigenes may serve as a source for novel gene discovery.

**Figure 3:**
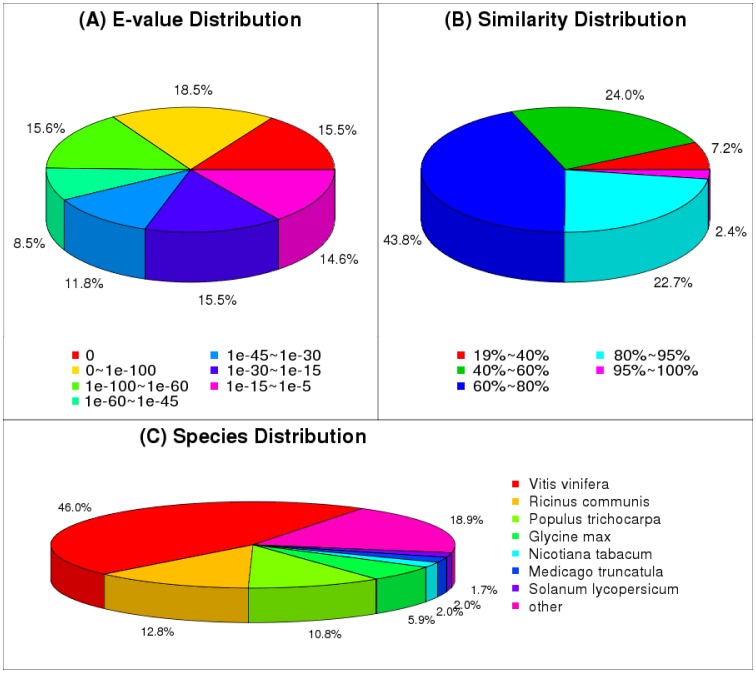
E-value Distribution. (A) similarity Distribution (B) and species distribution (C) of the BLAST matches of the transcriptome unigenes. This figure shows the distributions of unigenes BLASTX matches against the nr protein database (cutoff value E <1.0E-5) and the proportions for each species.

Based on the Nr annotations, we determined that 34.0% of the mapped unigenes had very significant homology to known sequences (E-value <10–100), 35.9% showed significant homology (10–100<E-value <10–30), and 30.1% showed weak homology (E-value 10–30 to 10–5) (Figure 3A); 25.1% of unigenes shared >80% similarity with known sequences, 67.8% shared between 40% and 80% similarity, and 7.2% shared 19% to 40% similarity (Figure 3B). Further, the unigenes shared strong homology with sequences from *Vitis vinifera* (46.0%), *Ricinus communis* (12.8%), *Populus trichocarpa* (10.8%), *Glycine max* (5.9%), and *Nicotiana tabacum* (2.0%) (Figure 3C).

### COG functional categories

**Figure 4:**
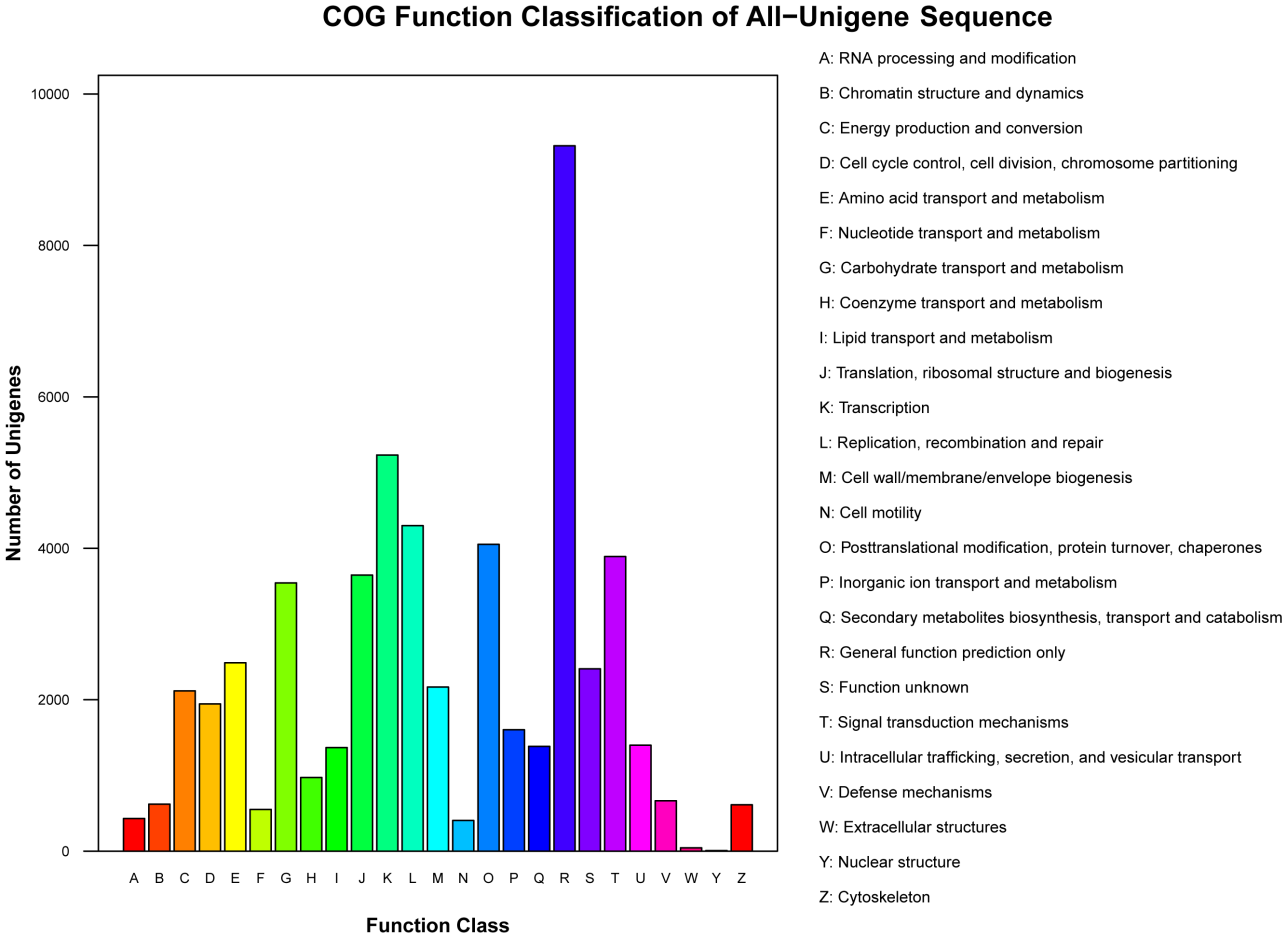
Classification of the clusters of orthologous groups (COG) for the transcriptome of *P. tomentosa* × *P. fortune.*

When the translated protein sequences of the unigenes were searched against the COG database, 28,165 unigenes (28.55%) were assigned to 25 function categories. The largest category was “General function prediction only” (9,316, 9.44%), followed by “Transcription” (5,232, 5.30%) and “Replication, recombination and repair” (4,299, 4.35%), while “Extracellular structures” (44, 0.045%) and “Nuclear structure” (8, 0.0081%) were the smallest categories (Figure 4).

### GO annotations

**Figure 5:**
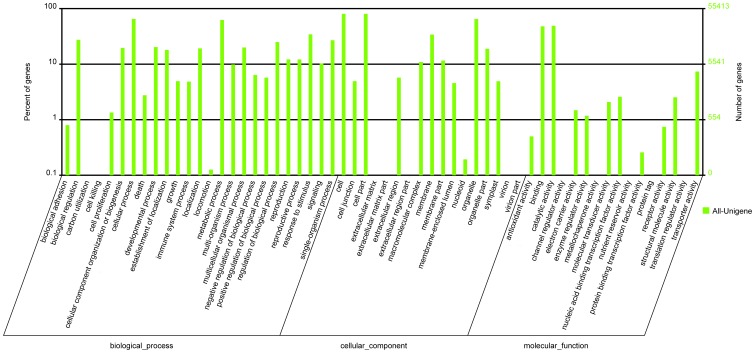
Classification of the gene ontology (GO) for the transcriptome of *P. tomentosa* × *P. fortune*.

The GO classification system was used to assign possible functions to the unigenes based on the annotations in Nr. A total of 55,413 unigenes (56.16%) were categorized into the three main GO categories (biological process, cellular component, and molecular function) and 44 functional groups. The largest groups under biological process were “cellular process” (36,028, 36.51%), “metabolic process” (34,472, 34.94%), and “response to stimulus” (19,059, 19.32%). Under cellular component, “cell” and “cell part” were the two largest groups, both containing 44,582 unigenes (45.18%). Under molecular function, the top three groups were “catalytic activity” (27,131, 27.50%), “binding” (26,550, 26.90%), and “transporter activity” (4,071, 4.12%) (Figure 5).

### KEGG pathways

To identify the different biochemical pathway that the annotated unigenes were associated with, we assigned the Enzyme Commission numbers (EC numbers) in the KEGG pathways. A total of 41,206 unigenes (approximately 41.76%) were mapped to 128 KEGG pathways, the top four of which were “Metabolic pathways”(ko01100, 10,041 unigenes, 24.37%), “Biosynthesis of secondary metabolites” (ko01110, 4,681 unigenes, 11.36%), “Plant-pathogen interaction” (ko04626, 2,284 unigenes, 5.54%), and “Plant hormone signal transduction” (ko04075, 2,089 unigenes, 5.07%), while “Betalain biosynthesis” (ko00965, 3 unigenes, 0.01%) was the least represented (Table S3).

### Transcriptional profiling of PTF2 and PTF4 under drought stress

**Figure 6:**
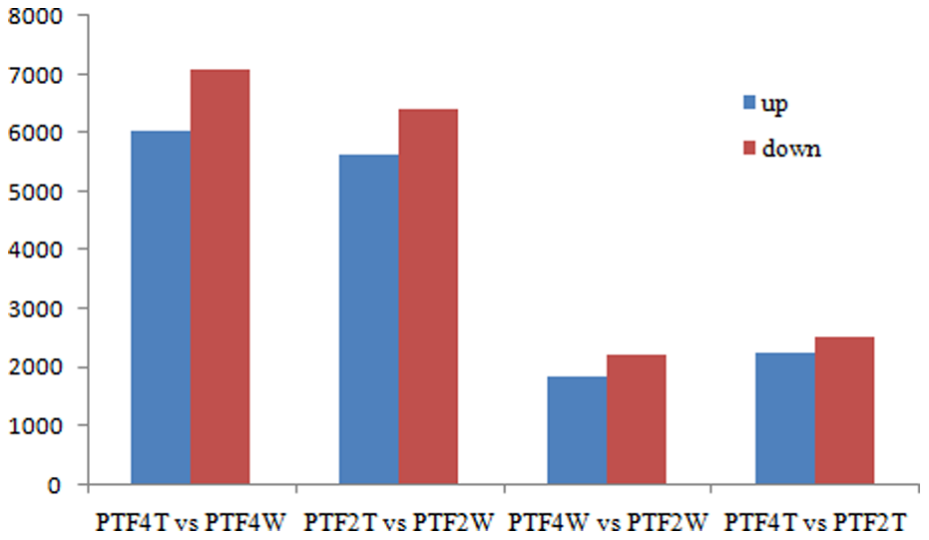
Statistics of differentially expressed genes in each pairwise comparison. Red bars represent the up-regulated genes while blue bars represent the down-regulated ones. PTF4T, 20 days drought treated tetraploid. PTF4W, well-watered tetraploid. PF2FT, 20 days drought treated diploid. PTF2W, well-watered diploid.

To identify transcriptional changes under drought stress, we performed four pairwise comparisons of the transcriptomes as follows: drought-treated PTF2 plants versus well-watered PTF2 plants (PTF2T vs. PTF2W), and drought-treated PTF4 versus well-watered PTF4 plants (PTF4T vs. PTF4W), well-watered PTF4 plants versus well-watered PTF2 plants (PTF4W vs. PTF2W), and drought-treated PTF4 versus drought-treated PTF2 plants (PTF4T vs. PTF2T). Large numbers of DEUs were detected (Table S4, S5, S6 and S7). The results of the pairwise comparisons of the DEUs are shown in Figure 6. Co-regulated DEUs between the PTF2T vs. PTF2W and PTF4T vs. PTF4W (the drought-treated vs. the well-watered) libraries were identified; 1,616 DEUs were up-regulated and 3,457 DEUs were down-regulated in the two comparisons. Similarly, co-regulated DEUs between the PTF4W vs. PTF2W and PTF4T vs. PTF2T (the tetraploid vs. the diploid) libraries were also identified; 173 DEUs were up-regulated and 98 DEUs were down-regulated in the two comparisons. Cell wall genes, photosynthesis-related genes, and genes encoding drought stress-related kinases, reactive oxygen species (ROS) scavenging enzymes, and osmotic adjustment proteins were among the most abundant transcripts. Some of the DEUs had no matches to known proteins; however, they may be found to contribute to drought tolerance when studied further (Table S8 and S9).

**Figure 7:**
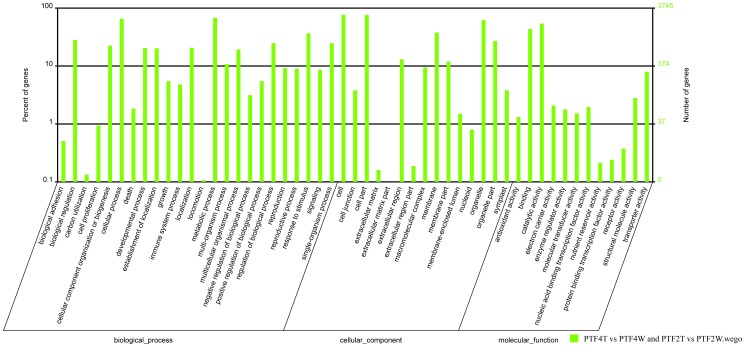
GO function analysis results for the consistently differentially expressed genes in both the PTF4T vs. PT4W and PTF2T vs. PT2W comparisons. PTF4T, 20 days drought treated tetraploid. PTF4W, well-watered tetraploid. PF2FT, 20 days drought treated diploid. PTF2W, well-watered diploid.

**Figure 8:**
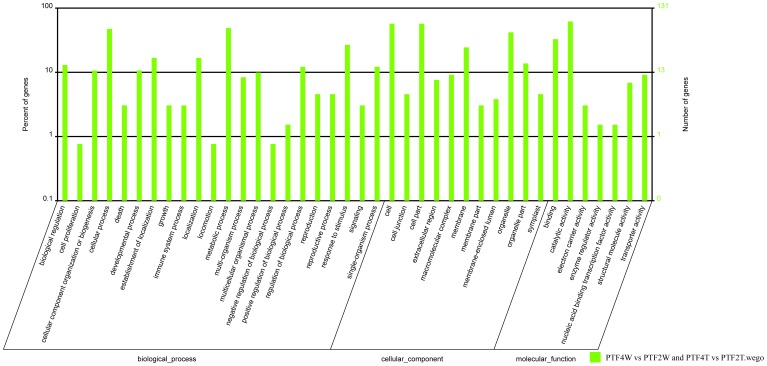
GO function analysis results for the consistently differentially expressed genes in both the PTF4W vs. PTF2W and PTF4T vs. PTF2T comparisons. PTF4W, well-watered tetraploid. PTF2W, well-watered diploid. PTF4T, 20 days drought treated tetraploid. PF2FT, 20 days drought treated diploid.

GO assignments allowed us to determine the major biological process, cellular component, and molecular function with which DEUs were associated (Figure 7 and 8). We then performed a GO enrichment analysis to identify the functional categories that were significantly enriched among the DEUs. Under biological process, stress-responsive processes (e.g., response to water deprivation, response to biotic stimulus, response to hormone stimulus, response to abiotic stimulus, and) were more represented in the up-regulated than in down-regulated DEUs, suggesting an increase of the related activities during drought treatment. Additionally, as reported previously in drought-stressed *P. euphratica* [50], GO terms like signal transduction, response to osmotic stress, small molecule metabolic process, carbohydrate catabolic process, arginine and proline metabolism, maltose metabolic process, disaccharide metabolic process, glucose catabolic process, hexose catabolic process, monosaccharide catabolic process, oligosaccharide metabolic process, and the related MAP kinase kinase activity were enriched. These findings may suggest that the osmotic stress-activated MAPK pathway and osmotic adjustment were crucial in the responses of PTF2 and PTF4 plants to water deprivation. Under cellular component, cell wall and plant-type cell wall were significantly enriched. Under molecular function, catalytic activity and binding were highly enriched for the drought-treated vs. the well-watered and the tetraploid vs. the diploid expression profiles, suggesting that the activities of many genes may be modulated in response to drought stress [51].

The co-regulated DEUs were also mapped to the KEGG metabolic pathways. Thirty-nine and seven metabolic pathways were significantly enriched (Q-value ≤0.05) in the drought-treated vs. the well-watered and the tetraploid vs. the diploid, respectively (Table S10 and S11).

### qRT-PCR verification of genes related to drought response in Paulownia leaves

**Figure 9:**
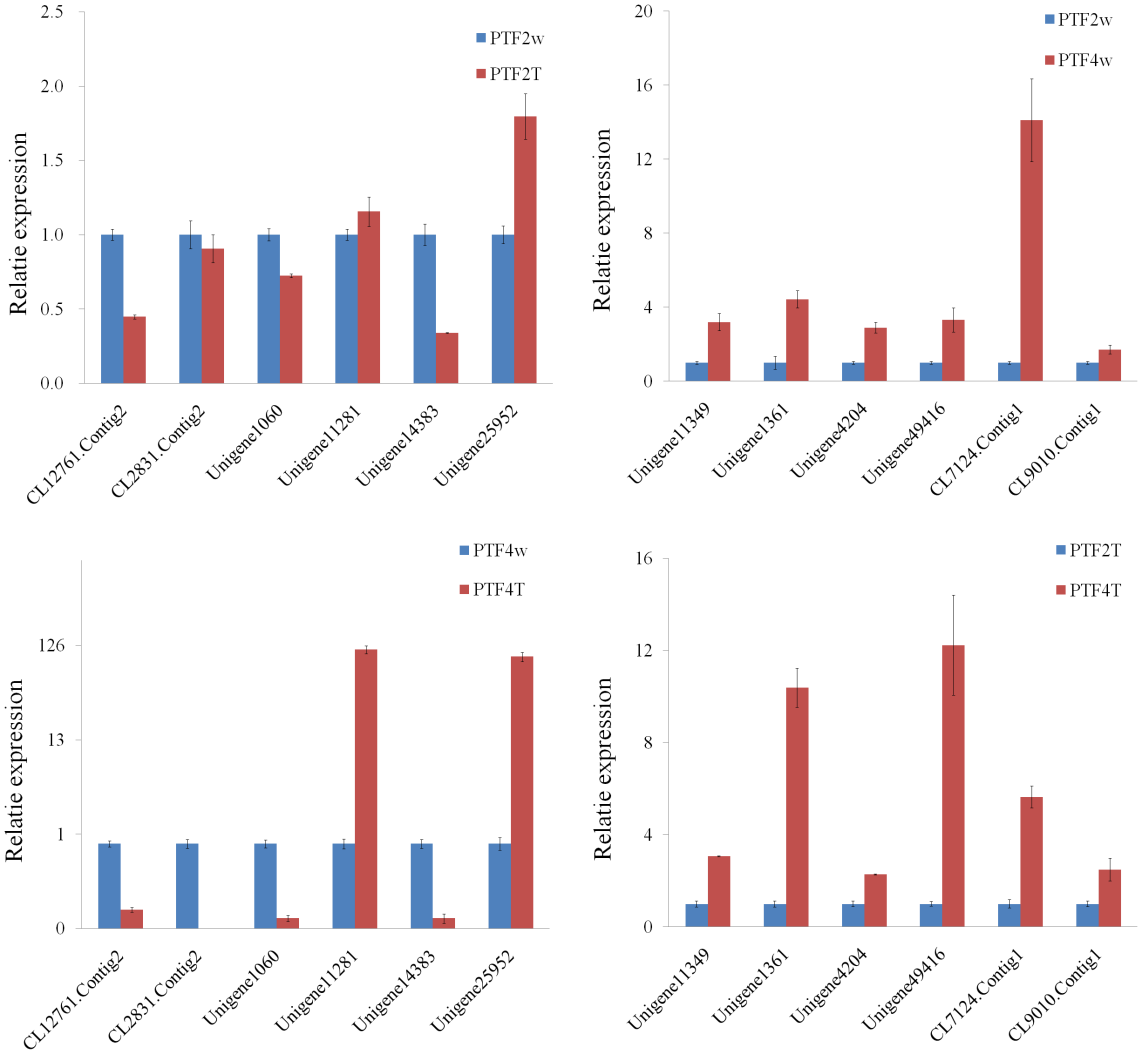
qRT-PCR analysis of candidate drought response genes. A, PTF2W, well-watered diploid; PTF2T, drought treated diploid. B, PTF4W, well-watered diploid; PTF4T, drought treated diploid.

The expression profiles of 13 DEUs from leaves of the drought-treated and the well-watered plants were assessed by qRT-PCR. The results showed that except for the mannose-1-phosphate guanyl transferase beta gene in the PTF2 plants, the expressions of all the other genes were consistent with the expressions from the transcriptome analyses (Figure 9).

## Discussion

Water deficiency significantly decreased plant growth in terms of height and basal stem diameter in the PTF2 and PTF4 plants. Paulownia is regarded as a relatively drought-tolerant species, and *P. tomentosa × P. fortune* plants, which were established in an ecosystem characterized by drought, have evolved special mechanisms that allow them to deal with insufficient water supply. In this study, the PTF4 plants exhibited more significant growth than the PTF2 plants in both the drought-treated and the well-watered samples. We also observed that PTF2 and PTF4 plants showed substantial differences in several physiological parameters in response to drought stress. The PTF4 seedlings exhibited higher relative water content (RWC), chlorophyll content, superoxide dismutase (SOD) activity, proline content and soluble protein content than the PTF2 seedlings under drought stress, this may indicate that PTF4 has stronger water absorption and cell turgor, and a more efficient enzymatic antioxidant system under water deficit conditions. Additionally, PTF4 seedlings showed lower malondialdehyde (MDA) content and relative electrical conductivity (REC) than the PTF2 seedlings in response to drought stress, suggesting more damage to membranes in PTF2 plants. These physiological parameters imply that PTF4 plants may be better capable of surviving drought conditions, which is consistent with the findings of our previous study [27]. In the present study, the transcriptome-wide gene expression profiling revealed differences in the abundance of drought-responsive genes between the PTF2 and PTF4 plants (Table S4, S5, S6, and S7). Under control conditions, transcript abundances of the majority of DEUs co-up-regulated in PTF2 plants were higher than those in PTF4 plants. However, under water-stress conditions, transcript abundances of co-up-regulated DEUs significantly increased in PTF4, but they maintained a relatively stable level in PTF2 plants, especially DEUs involved in ROS-scavenging system, amino acid and carbohydrate metabolism, and plant hormone biosynthesis and transduction (Table S8 and S9). This implies that PTF4 plants had a greater capability to tackle drought stress by increasing abundance of metabolism and defense-related unigenes. GO terms and pathways analysis showed that diverse metabolic processes were altered and some DEUs indicated the possibility of crosstalk between pathways in the plants during drought stress. These changes might help the PTF2 and PTF4 plants survive under drought conditions.

### Protection from oxidative damage

ROS accumulate in chloroplasts and produce a leakage of electrons towards oxygen under water deficit conditions. ROS serve as integral cellular signal molecules in stress sensing/signal transduction processes; however, excessive levels of ROS may induce oxidative damage to proteins, DNA, and lipids [52]. To protect tissues from oxidative damage, plants have developed an enzymatic antioxidant defense system to remove excess ROS. The ROS-scavenging system of plants includes several important antioxidant enzymes such as superoxide dismutase and ascorbate peroxidase [53]. In the present study, major ROS-scavenging enzymes and antioxidants including superoxide dismutase, ascorbate peroxidase, glutathione peroxidase, and lactoylglutathione lyase were up-regulated by drought stress (Table S8). However, differences in the expressions of these antioxidant genes were also observed between the two accessions, and the fold changes of these unigenes in the SOD, APX, and CAT families were almost always greater in the PTF4 plants than that in the PTF2 plants (Table S9). This is supported by the observation that both PTF2 and PTF4 plants showed increasing SOD activity, and the values of this trait in the PTF4 plants were always at much higher levels than in the PTF2 plants over the entire treatment process.

A senescence-associated protein was up-regulated in the PTF2 and PTF4 plants under drought stress, and its homologs, senescence associated protein 1 and senescence-associated gene 21, were over-expression in Arabidopsis to protect the plant from oxidative damage [54]. The Unigene22025 transcript was strongly enriched and up-regulated in the PTF2 and PTF4 plants during drought stress; interestingly, this transcript was also reported to be significantly differentially expressed in a previous study [55]. Additionally, the PTF2 and PTF4 plants were found to accumulate of high amounts of galactinol and raffinose during drought stress (discussed below), which may also help improve ROS scavenging in the drought-stressed plants [56].

### Amino acid and carbohydrate metabolism

Low-molecular weight compatible osmolytes (osmoprotectants), such as proline, glycine, betaine, and sugar alcohols have been reported to accumulate in a variety of plant species in response to environmental stresses. This accumulation allows plants to maintain their water absorption and cell turgor under water deficit conditions by allowing rapid water intake from rather dry soil, thereby, mitigating the effects of water stress [57]–[59]. In the present study, various genes that may be involved in proline and carbohydrate metabolism were found.

Proline is a common osmoprotectant in water-stressed plants and its dramatic accumulation has been shown to confer osmotic tolerance to plants during drought stress [60]. In our study, unigenes encoding aldehyde dehydrogenases and glutamate synthase, which play important roles in catalyzing the conversion of Δ^1^-pyrroline 5-carboxylate synthetase (P5C) to glutamate, were found to be down-regulated in the PTF2T and PTF4T plants; however, the expression levels of these DEUs were higher in PTF2T than in PTF4T. Conversely, a unigene encoding proline dehydrogenase (ProDH) was up-regulated in PTF2T, while its expression was unaltered in PTF4T plants (Table S8 and S9).

In plants, glutamate is catalyzed to P5C by Δ^1^-pyrroline 5-carboxylate synthetase (P5CS) and P5C is then converted to proline by P5C reductase (P5CR). However, ProDH catalyzes the conversion of proline to P5C [61]. Therefore, the accumulation of proline in dehydrated plants has been linked to the up-regulation of P5CS and the down-regulation of ProDH [62]. In our study, unigenes encoding P5CS and P5CR were not among the selected DEUs (Table S8 and S9). However, the values of physiological parameters showed that the proline content in the PTF4 plants increased significantly with the increase of the drought stress, we therefore speculate that processes other than transcriptional regulation might play a role in influencing the accumulation of proline in PTF4 plants.

Unigenes encoding proteins involved in carbohydrate metabolism (e.g., galactinol, sucrose, fructose, and mannose metabolism) accounted for a large number of the DEUs. It has been reported that the accumulation of soluble carbohydrates is strongly correlated with stress tolerance in plants [63]. Under drought stress, we observed that most of these DEUs involved in carbohydrate metabolism were co-up-regulated in the PTF4 and PTF2 plants; however, the fold changes of these unigenes were greater in PTF4 than in PTF2 plants (Table S8 and S9). The expression of the unigene encoding galactinol synthase (GolS), which is involved in galactinol and raffinose family oligosaccharides (RFO) metabolism, was increased in PTF2T and PTF4T plants. In Arabidopsis, the homologs of *GolS, AtGolS1* and/or *AtGolS2*, were reported to be over-expressed, which increased galactinol and raffinose accumulation and enhanced the plant’s tolerance to drought stress and oxidative damage [56]. The GolS gene in *Populus balsamifera* was found to be significantly differentially expressed in response to drought stress [64]. GolS catalyzes the first step in the biosynthetic pathway of galactinol and raffinose family oligosaccharides using galactose and myo-inositol as substrates. Several unigenes encoding aldose 1-epimerase, which are involved in galactose and myo-inositol synthesis, were found to be elevated in both PTF4T and PTF2T plants (Table S8 and S9). The expressions of several trehalose synthase encoding unigenes were found to be significant enhanced in response to drought stress in both accessions. The trehalose-6-phosphate synthase 1 (TPS1) encoding gene, which was homologous to *AtTPS1,* was of particular interest because *AtTPS1* plays an important role in abscisic acid (ABA) and sugar signaling and its over-expression resulted in improved drought tolerance in Arabidopsis [65]. Further, the over-expression of *AtTPS1* in rice was reported to elevate the sucrose, fructose, and glucose content and enhance the drought stress response [66]. Several DEUs with homology to glucose-6-phosphate/phosphate translocator 2 (GPT2), which was found to be involved in glucose 6-phosphate transport in Arabidopsis [67], were up-regulated in PTF4T and PTF2T, implying that the sugar signal triggered by GPT2 may be important in regulating drought stress tolerance in *P. tomentosa* × *P. fortunei* plants. We also found a very robust increase in soluble sugar contents that was more pronounced in PTF4 than PTF2 plants in our physiological measurements. The accumulation of these osmoprotectants in the leaves could help enhance the osmotic stress tolerance of *P. tomentosa* × *P. fortunei.*

### Hormone biosynthesis

Plant hormones are closely related to plant growth and stress tolerance [68], [69]. ABA plays crucial roles in various stress responses and the accumulation of endogenous ABA is known to be associated with drought tolerance [70], [71]. Recently, several studies have revealed the molecular basis of ABA biosynthesis and its response mechanisms [72]–[74].

The over-expression of 9-cis-epoxycarotenoid dioxygenase (NCED), Zeaxanthin epoxidase (ABA2), and phytoene synthase were reported to be associated with the conversion of zeaxanthin to ABA, which can be metabolized and catalyzed by the ABA hydroxylase CYP707A1 [75]. In transgenic Arabidopsis plants, *AtNCED3* and the phytoene synthase gene were induced by drought and their up-regulation was reported to enhance drought tolerance by increasing the endogenous ABA level [76], [77]. Furthermore, ABA2 was found to be a negative regulator of ABA in Arabidopsis [78], [79]. Therefore, ABA biosynthesis can be enhanced by positive regulators (NCED3, phytoene synthase) and inhibited by negative regulators (ABA2). In our study, transcript abundances of hormone biosynthesis-related DEUs were significantly different between PTF2 and PTF4. The unigenes that were most similar to the Arabidopsis *NCED3* and phytoene synthase gene sequences were up-regulated, and the unigenes encoding the ABA2 enzymes were down-regulated in the PTF2T and PTF4T plants, transcript abundances of these co-up-regulated and co-down-regulated DEUs have higher fold change in PTF4 than in PTF2 (Table S8 and S9). Our data suggest that these genes may play important roles in regulating the biosynthesis of ABA in the two accessions.

ABA signaling pathways have been linked to phospholipase D (PLD), protein phosphatase 2C (PP2C), SNF1-related kinases (SnPKs), and CBL-interacting protein kinases (CIPKs), and unigenes encoding these enzymes were significantly up- or down-regulation more in the PTF4T than that in the PTF2T plants under drought stress (Table S8 and S9). PP2C was found previously to negatively regulate ABA signaling in Arabidopsis [80] and links between PP2C and SnRK2 have been reported [81]–[83]. Several members of the SnRK2 family in Arabidopsis and rice were shown to be activated in response to ABA or osmotic stress [84], [85], and in fava bean guard cells SnRK2 was shown to play an important role in ABA-dependent stomatal closure [86]. Together these results suggest that SnRK2 is an important regulator of ABA signal transduction during drought stress [83]. CIPKs and their interacting partner calcineurin B-like (CBL) (also known as SnRK3) form a complex that was reported to be a central hub of the ABA-mediated signaling pathway under drought, cold, or salt stress [87], [88]. Although several genes encoding potential factors of the ABA signaling pathway were identified in the PTF2T and PTF4T plants, the molecular mechanisms that control this complex signaling pathway in *P. tomentosa × P. fortunei* need to be investigated further.

Recent studied have revealed that the plant hormone indol-3yl-acetic acid (IAA) plays a critical role in regulating plant growth and cell enlargement [89]. The auxin transporter protein AUX1 has been found to be a major influx carrier [90]. Aux/IAA proteins and the ARF transcription factors are important regulators of IAA-modulated gene expression, and auxin-responsive promoter (GH3) and auxin-responsive protein (SAUR) have been reported to regulate plant growth and cell enlargement [89], [91]. In the present study, several increased transcript abundance of the DEUs in both PTF2T and PTF4T were annotated as being involved in crosstalk among the IAA encoding unigenes, whereas we observed that the expression level of these DEUs were higher in PTF4 under drought stress (Table S8 and S9), suggesting that different levels of IAA accumulation might explain the cell enlargement and plant growth differences observed between the PTF2 and PTF4 plants.

### Multi-stress responsive genes in *P. tomentosa* × *P. fortunei*

Transcription factors (TFs) have been shown to be critical upstream regulators of plant responses to various biotic and abiotic stresses [92]. In the present study, a large number of DEUs encoded TFs belonging to the MYB/MYC, ABI3VP1, AP2-EREBP, DREB, ARF, bzip, ABA responsive, NAC, WRKY, bHLH, ZF-HD, AREB/ABF, and ZFP families. Under control conditions, most of the DEUs encoded TFs had higher transcript abundance in PTF2 than in PTF4, while under drought stress, expression changes of these DEUs were significantly higher in PTF4 than in PTF2 plants (Table S8 and S9). These TFs were divided into ABA-dependent signaling systems and ABA-independent signaling pathways. Over-expression of the MYB/MYC, bHLH, NAC, WRKY, ZFP, bzip, and ZF-HD families has been shown to play significant roles in controlling the expression of specific stress-related genes and plant stress signal transduction, thereby greatly enhancing the tolerance of plants to drought and high salinity in Arabidopsis and rice [93]–[97]. Recently, transcriptional activators that up-regulate stress-responsive genes have been used widely to impart drought tolerance to transgenic plants [98]. The over-expression of the AP2-type TF and ethylene-responsive transcription factors (SHN1–3) in transgenic plants were found to regulate several stress inducible target genes and increase drought tolerance [99]. Increased expression of the NAC transcription factor 45 (OsNAC045) in transgenic rice plants was found to enhance drought tolerance, which may serve as a useful tool for improving drought stress response in genetically engineered rice [94]. Recent studies have shown that ABRE-binding bZIP and OsABF2 were detectable in transgenic rice where they positively regulated drought tolerance and ABA signaling [100]. Transcriptional repressors that have the ability to repress the expression of genes under drought stress have been shown to be important in engineering drought tolerance in plants [101]. AtMYB60 was reported to adjust stomatal movement and function as a transcriptional repressor in the plants response to dehydration; specifically, the expression of this gene in Arabidopsis guard cells was negatively regulated during drought stress [102]. A null T-DNA insertion mutation in the AtMYB60 gene led to reduced stomatal opening and minimized wilting during drought stress, this mutation resulted in defective guard cell but had no significant effect on other physiological processes and agricultural traits [102]. In our study, several signal transduction pathways that independently respond to drought stress (in both an ABA dependent- and independent- manner) were identified in the PTF2T and PTF4T plants. ABA-dependent signaling pathway have been illustrated that they could mediate stress adaptation by the action of ABA-inducible TFs controlling the expression of genes containing cis-acting ABA response elements (e.g., TFs such as MYB/MYC and AREB/ABF), while ABA-independent stress signaling pathway leading to gene expression involve different TFs such as (1) NAC and ZF-HD, and (2) CBF/DREB (Figure S2) [103]–[106], suggesting that drought stress tolerance at the transcriptional level was controlled by an extremely intricate gene regulatory network. Thus, functional analysis of drought inducible TFs will help in the development of drought tolerant plants that will survive well in acute field condition.

Different sets of genes are known to be associated with specific stress-responsive signal transduction. Mitogen-activated protein kinases (MAPKs) are a highly conserved family of serine/threonine protein kinases that are involved in a variety of fundamental cellular processes such as proliferation, differentiation, stress response, and survival. The MAPK cascade has been found to lead to accumulated production of osmolytes by activating, for example, the protein tyrosine kinases and G-protein receptors [107], [108]. The MAPK pathway phosphorylates a three-tiered kinase cascade: MAPKKK→MAPKK→MAPK, which propagates downstream stress signals [109]. Activated MAPK can migrate to the nucleus to activate the TFs directly. These findings indicate that modulating MAPK may lead to altered expression of specific regulatory TFs and protein kinases towards enhanced drought tolerance in plants. In this study, many DEUs in the PTF2T and PTF4T plants were identified as MAPKs including MAPK8, MAPK9, MAPK14, MAPKK2, MAPKK6, MAPKKK1, and MAPKKK2, and a relatively large proportion of DEUs encoding MAPKs were up-regulated both in the PTF2 and PTF4 plants under drought stress, but the fold changes of these DEUs were greater in PTF4 than in PTF2. We also identified a great number of calcium-dependent protein kinases (CDPKs) that were activated and the expression levels of these unigenes were induced strongly in PTF4 plants under drought treatments (Table S8 and S9). Previous studies have indicated that CDPKs play a crucial role in Ca^2+^-mediated signal transduction in plants in response to drought and salinity stresses, suggesting that signal transduction may play a critical role in the *P. tomentosa × P. fortunei* drought response [107], [110].

Plants have been found to survive different stresses using multifunctional genes that enable crosstalk among the various abiotic stress resistance systems [111]. We also found that in PTF4, several stress-regulated unigenes like lipid transfer protein, late embryogenesis abundant protein (LEA), osmotin, small heat shock protein, HSP70, HSP90, antiporter, and dehydrins were expressed at higher transcript abundance than in PTF2 plants under drought stress. Several study reported that these genes were generally involved in cell rescue and defense mechanisms and it was suggested that their overexpression may play an important role in conferring dehydration and drought stress by causing stomatal closure and osmotic adjustment [112]–[116].

## Conclusions

PTF4 trees have high ecological and economic value. In the present work, we performed large-scale transcriptome sequencing of diploid and autotetraploid *P. tomentosa × P. fortune* plants under drought stress using an Illumina/Solexa sequencing platform. More than 291 million reads were generated from four libraries and *de novo* assembled into 98,671 nonredundant unigenes that will provide a useful resource for this species. GO terms enrichment and pathways analyses of the annotated DEUs revealed changes in several metabolic processes as a result of drought stress. These results may shed some light on the biology of the drought response and tolerance in Paulownia species. In addition to the previously reported drought stress related genes detected in the libraries, a large number of unigene sequences could not be mapped to known sequences in the protein databases. Some of the unidentified DEU sequences may be novel and/or unknown Paulownia genes associated with drought stress. Functional analysis of these genes may contribute to a deeper understanding of the response of Paulownia plants to drought. Our findings will help enrich our understanding of the drought response in diploid and autotetraploid *P. tomentosa × P. fortune* and may provide new insights into the responses of woody plants to drought stress.

## Supporting Information

Figure S1 **Distribution of unigene lengths in the transcriptome of** ***P. tomentosa*** **×** ***P. fortunei.*** The sizes of all unigenes were calculated.
(TIF)

Figure S2 **A schematic representation of transcriptional regulatory networks of cis-acting elements and transcription factors involved in abiotic-stress-responses.** Transcription factors are shown in ellipses; cis-acting elements are shown in boxes.
(TIF)

Table S1 **Primers of quantitative RT-PCR analysis of candidate drought response genes.** -f represents forward primers and -r represents reverse primers.
(DOCX)

Table S2 **Unigene annotations based on their BLASTX hits in the nr database.**
(XLSX)

Table S3 **KEGG annotation of unigenes.**
(XLSX)

Table S4 **Differetially expressed genes in the PTF2T vs. PTF2W comparison.** PTF2T, 20 days drought-treated diploid; PTF2W, well-watered diploid.
(XLSX)

Table S5 **Differetially expressed genes in the PTF4T vs. PTF4W comparison.** PTF4T, 12 days drought-treated tetraploid; PTF4W, well-watered tetraploid.
(XLSX)

Table S6 **Differetially expressed genes in the PTF4W vs. PTF2W comparison.** PTF4W, well-watered tetraploid; PTF2W, well-watered diploid.
(XLSX)

Table S7 **Differetially expressed genes in the PTF4T vs. PTF2T comparison.** PTF4T, 20 days drought-treated tetraploid; PTF2T, 20 days drought-treated diploid.
(XLSX)

Table S8 **Consistently differentially expressed genes in both the PTF2T vs. PTF2W and PTF4T vs. PTF4W comparisons.** PTF2T, 20 days drought-treated diploid; PTF2W, well-watered diploid; PTF4T, 20 days drought-treated tetraploid; PTF4W, well-watered tetraploid.
(XLSX)

Table S9 **Consistently differentially expressed genes in both the PTF4W vs. PTF2W and PTF4T vs. PTF2T comparisons.** PTF4W, well-watered tetraploid; PTF2W, well-watered diploid; PTF4T, 20 days drought-treated tetraploid; PTF2T, 20 days drought-treated diploid.
(XLSX)

Table S10 **KEGG pathway analysis results for the consistently differentially expressed genes in both the PTF2T vs. PTF2W and PTF4T vs. PTF4W comparisons.** PTF2T, 20 days drought-treated diploid; PTF2W, well-watered diploid; PTF4T, 20 days drought-treated tetraploid; PTF4W, well-watered tetraploid.
(XLSX)

Table S11 **KEGG pathway analysis results for the consistently differentially expressed genes in both the PTF4W vs. PTF2W and PTF4T vs. PTF2T comparisons.** PTF4W, well-watered tetraploid; PTF2W, well-watered diploid; PTF4T, 20 days drought-treated tetraploid; PTF2T, 20 days drought-treated diploid.
(XLSX)
