# Supplementary figures and images for: Transcriptome-Wide Profiling and Expression Analysis of Diploid and Autotetraploid Paulownia tomentosa × Paulownia fortunei under Drought Stress
Source: PLoS One. 2014 Nov 18;9(11):e113313. doi: 10.1371/journal.pone.0113313 (PMC4236183; doi:10.1371/journal.pone.0113313)

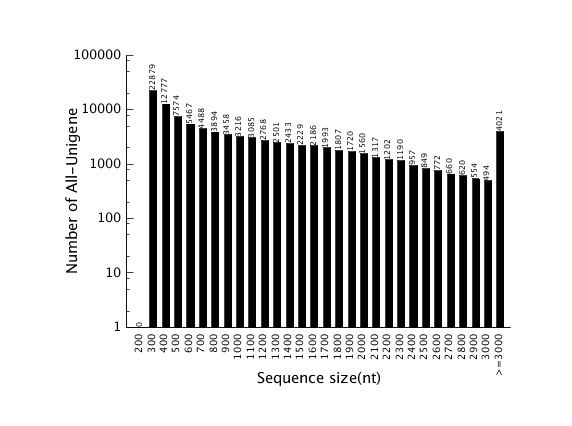

Supplement: Figure S1 — Distribution of unigene lengths in the transcriptome of P. tomentosa × P. fortunei. The sizes of all unigenes were calculated. (TIF) [file pone.0113313.s001.tif]

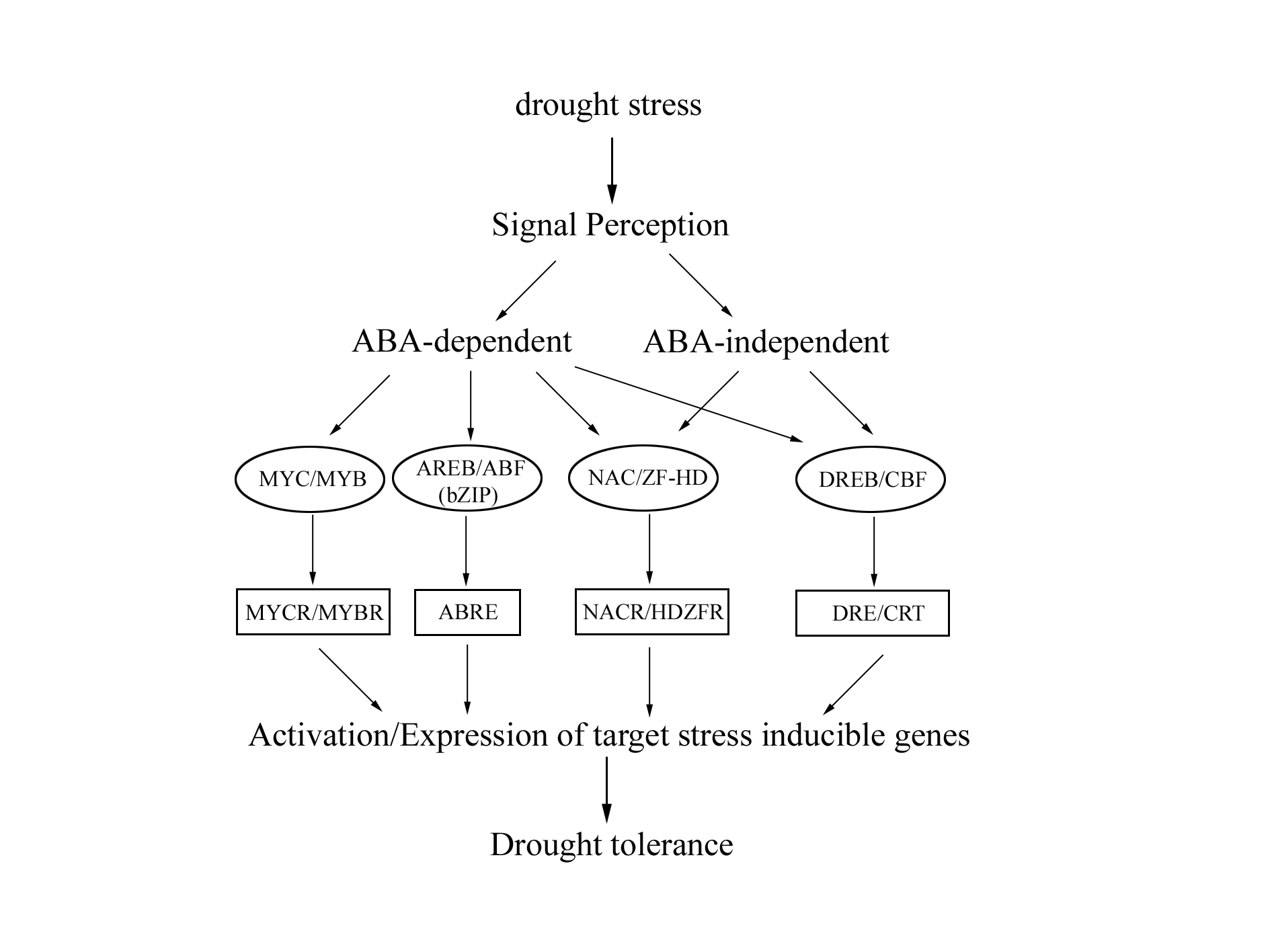

Supplement: Figure S2 — A schematic representation of transcriptional regulatory networks of cis-acting elements and transcription factors involved in abiotic-stress-responses. Transcription factors are shown in ellipses; cis-acting elements are shown in boxes. (TIF) [file pone.0113313.s002.tif]
