# Supplementary material for: Transcriptome-Wide Profiling and Expression Analysis of Diploid and Autotetraploid Paulownia tomentosa × Paulownia fortunei under Drought Stress
Source: PLoS One. 2014 Nov 18;9(11):e113313. doi: 10.1371/journal.pone.0113313 (PMC4236183; doi:10.1371/journal.pone.0113313)
Supplement: Table S1 — Primers of quantitative RT-PCR analysis of candidate drought response genes. -f represents forward primers and -r represents reverse primers. (DOCX) [file pone.0113313.s003.docx]

**Supplemental Table 1 Primers of quantitative RT-PCR analysis of candidate drought response genes** -f represents forward primers and -r represents reverse primers.

| Potential gene function | Size (nt) | Primer | Sequence |
| --- | --- | --- | --- |
| GDSL esterase/lipase | 1092 | Unigene11349_All-f | CTTCGTCTACTGCCATTC |
|  |  | Unigene11349_Alll-r | GTTGTTGTTGCCATTATCC |
| hypothetical protein | 480 | Unigene1361_All-f | CTCAGCATCCCAAGTCTCC |
|  |  | Unigene1361_All-r | GCCTTCGTAGTTGTTGTAGC |
| Protein phosphatase 2C | 1164 | Unigene14383_All-f | AGAGATGGAGGATGATGTTG |
|  |  | Unigene14383_All-r | CAGTGCCTTCTTGATTGC |
| Laccase-20 | 1725 | Unigene4204_All-f | GCACAATCACCTTCCTCAG |
|  |  | Unigene4204_All-r | GTGTATGTTGGCGTAGACC |
| osmotin-like protein | 741 | Unigene11281_All-f | CACTCTCCATCCTCCTTATCC |
|  |  | Unigene11281_All-r | GTTGTTGACGACTGTTAGAATG |
| Proline-rich receptor-like protein kinase PERK9 | 705 | Unigene49416_All-f | ACCGAGGAGGAGAACAAG |
|  |  | Unigene49416_All-r | CAATCAACTAGGCATACTAAGC |
| solute carrier family 39 | 1050 | CL7124.Contig1_All-f | GCAGCAAGCAAGCACATTAC |
|  |  | CL7124.Contig1_All-r | GAAGGCGAAGGTGGAAGC |
| galactose-1-phosphate uridyltransferase | 672 | CL9010.Contig1_All-f | TCAGAGATTACACGACGATACG |
|  |  | CL9010.Contig1_All-r | GAACTTGCGGACGGTAGC |
| Auxin-responsive protein IAA26 | 837 | Unigene25952_All-f | GGGTGTCGTCAGTTTATGGATTTG |
|  |  | Unigene25952_All-r | GGACCAAGCCTCAACTCAAGC |
| chloroplast PsbO4 precursor | 990 | CL12761.Contig2_All-f | TGACTACGCTGCTGTTACTG |
|  |  | CL12761.Contig2_All-r | GGATGACCCTCTGTATGATGG |
| chloroplast ferredoxin-NADP+ oxidoreductase precursor | 1089 | Unigene1060_All-f | TGCCTTCCTCCAAGTCTG |
|  |  | Unigene1060_All-r | GATACCTTCTCAGCCTTAGC |
| chloroplast sedoheptulose-1,7-bisphosphatase | 1176 | CL2831.Contig2_All-f | ATATGTTCTCGCTCTCAAGG |
|  |  | CL2831.Contig2_All-r | TCGCAAGGTGTATTTCTCC |
